# The application value of CT radiomics features in predicting pressure amplitude correlation index in patients with severe traumatic brain injury

**DOI:** 10.3389/fneur.2022.905655

**Published:** 2022-08-25

**Authors:** Jiaqi Liu, Yingchi Shan, Guoyi Gao

**Affiliations:** ^1^Department of Neurosurgery, Shanghai General Hospital, Shanghai Jiao Tong University School of Medicine, Shanghai, China; ^2^Department of Plastic and Reconstructive Surgery, Shanghai 9th People's Hospital, Shanghai Jiao Tong University School of Medicine, Shanghai, China

**Keywords:** traumatic brain injuries, intracranial pressure, computed tomography, non-invasive evaluation, texture features

## Abstract

**Purpose:**

To explore the application value of a machine learning model based on CT radiomics features in predicting the pressure amplitude correlation index (RAP) in patients with severe traumatic brain injury (sTBI).

**Methods:**

Retrospectively analyzed the clinical and imaging data in 36 patients with sTBI. All patients underwent surgical treatment, continuous ICP monitoring, and invasive arterial pressure monitoring. The pressure amplitude correlation index (RAP) was collected within 1 h after surgery. Three volume of interest (VOI) was selected from the craniocerebral CT images of patients 1 h after surgery, and a total of 93 radiomics features were extracted from each VOI. Three models were established to be used to evaluate the patients' RAP levels. The accuracy, precision, recall rate, F1 score, receiver operating characteristic (ROC) curve, and area under the curve (AUC) were used to evaluate the predictive performance of each model.

**Results:**

The optimal number of features for three predicting models of RAP was five, respectively. The accuracy of predicting the model of the hippocampus was 77.78%, precision was 88.24%, recall rate was 60%, the F1 score was 0.6, and AUC was 0.88. The accuracy of predicting the model of the brainstem was 63.64%, precision was 58.33%, the recall rate was 60%, the F1 score was 0.54, and AUC was 0.82. The accuracy of predicting the model of the thalamus was 81.82%, precision was 88.89%, recall rate was 75%, the F1 score was 0.77, and AUC was 0.96.

**Conclusions:**

CT radiomics can predict RAP levels in patients with sTBI, which has the potential to establish a method of non-invasive intracranial pressure (NI-ICP) monitoring.

## Introduction

Traumatic brain injuries (TBI) are a type of trauma with high morbidity, disability, and mortality rates (1). Currently, the treatment of patients with TBI is based on surgery combined with neurological intensive care (2, 3). Intensive care treatment is a very important part of the patient's treatment process, in which intracranial pressure (ICP) monitoring is necessary for neurocritical care treatment (3). In clinical work, ICP monitoring alone does not provide comprehensive information about the patient's intracranial pressure, and its derived parameters such as the relationship of amplitude and pressure (RAP) can more comprehensively and objectively reflect the patient's ICP level (4, 5). The RAP can reflect the changes in the ICP level of patients more comprehensively and objectively, which can assist physicians to make decisions and adjustments in patient treatment and to implement individualized medical treatment precisely (6).

The implementation of ICP monitoring relies on the surgical placement of an intracranial pressure monitoring probe, which is not yet in line with modern neurosurgery's pursuit of speed, accuracy, and efficiency. CT scanning is one of the most popular imaging techniques used in neurosurgery, and is fast, non-invasive, and efficient, thus playing a key role in neurosurgical care. In the past, clinicians used CT images to interpret the degree of displacement of intracranial structures to assess the ICP level of patients, but this method lacks theoretical support. The essence of this study is to obtain high-throughput, quantitative imaging features from CT images, filter the features, and build a reasonable prediction model through machine learning. The aim of this study is to explore the relationship between CT imaging histological features and RAP in patients with TBI, so as to reveal whether building a machine learning model based on CT imaging histological features can objectively and in real-time reflect ICP-related parameters of patients, and to explore the clinical application value of this machine learning model for realizing non-invasive ICP monitoring.

## Materials and methods

### Study design and setting

Clinical data of patients with sTBI admitted to the Department of Neurosurgery, Shanghai General Hospital from January 2019 to December 2020 were collected and analyzed. Inclusion criteria were as follows: (1) emergency admission due to closed craniocerebral injury with a clear history of trauma; (2) older than 18 years of age, younger than 65 years of age, regardless of gender; (3) received invasive ICP monitoring; (4) cranial CT was reviewed within 1 h after surgery. Exclusion criteria were as follows: (1) patients with a history of traumatic brain injury, cerebral infarction, brain tumor, or other neurological diseases or cranial surgical interventions that might result in an abnormal anatomical structure; (2) with previous coagulopathy and blood system related diseases. The study protocol conformed to the ethical guidelines of the Declaration of Helsinki, and this study was approved by the Ethics Committee of Shanghai General Hospital, Shanghai Jiao Tong University School of Medicine. Participants' right to know was fully guaranteed and indicated in the ethical approval document.

### Data sources and measurements

In addition to the baseline characteristics, we mainly collected and analyzed the cranial CT after the surgery and the RAP value recorded by a Neumatic data collector (a machine that could analyze the waveform of intracranial pressure). The CT-related features were acquired from the Digital Imaging and Communications in Medicine (DICOM) file of the last cranial CT before ICP monitoring using a 64-slice spiral CT machine (General Electric Medical Systems, USA). As per the routine protocol of a CT scan, the CT slices were parallel to the orbitomeatal plane from the foramen magnum to the vertex. The scanning slice thickness was 1 mm. Patients were returned to ICU after surgery. Radial artery puncture was performed in all patients, the arterial indwelling needle was connected to the baroreceptor, and the baroreceptor was connected to the bedside monitor, for continuous invasive arterial pressure monitoring. Finally, the Neumatic data collector was connected to the bedside monitor through a network cable port to collect real-time intracranial pressure-related parameters. All RAP data collected within 1 h after the start of monitoring (monitoring sampling frequency is 3 s, a total of 1,200 data in 1 h) were recorded, and the mean value of RAP was calculated as the analysis index.

**Figure 1:**
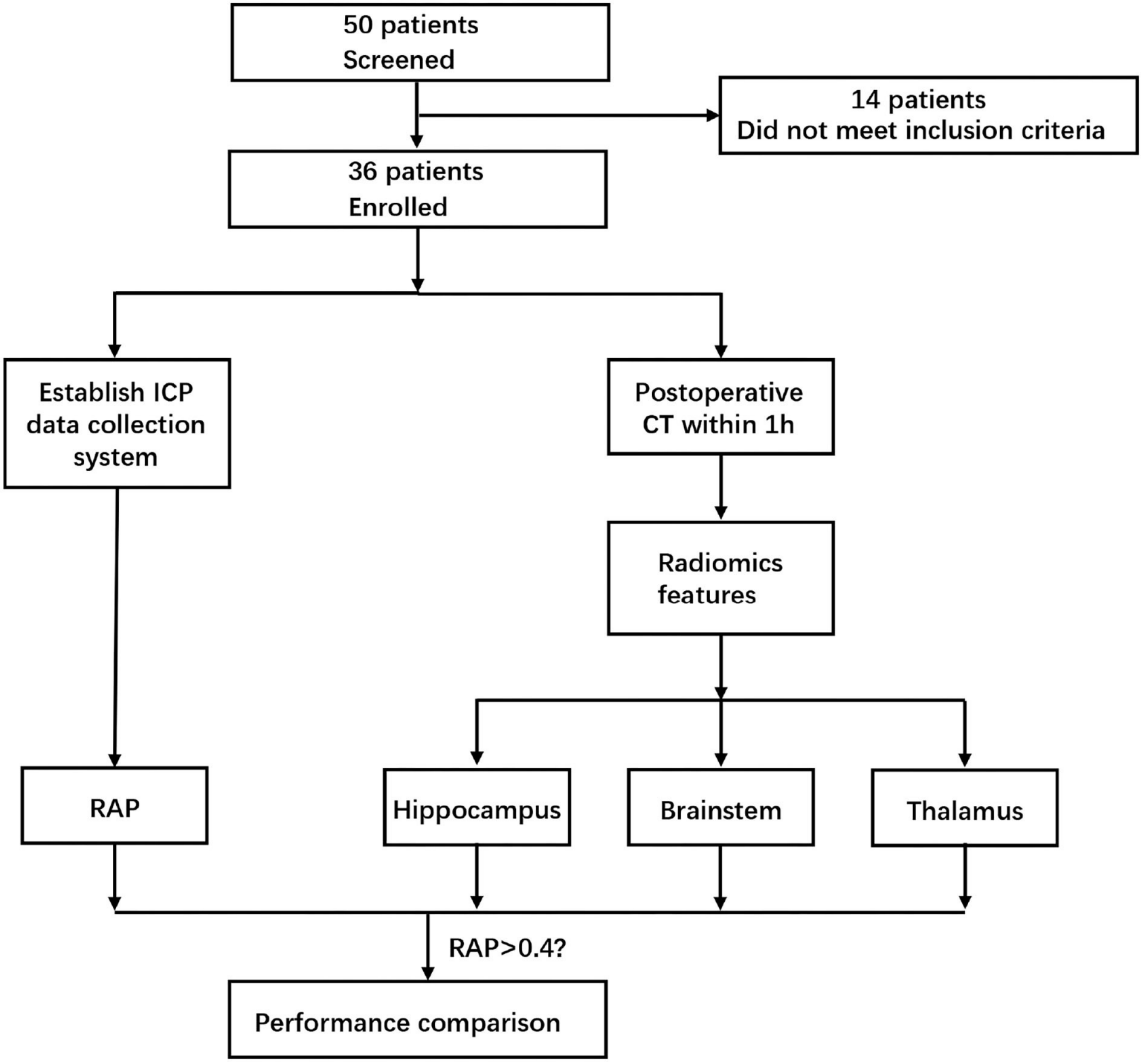
Case collection and data analysis process.

After obtaining the patient's CT scan within 1 h postoperatively, three VOI with the size of 5 ^*^ 5 ^*^ 5 mm were extracted from the hippocampal gyrus, brainstem, and thalamus of the injured side by pydicom module in the Python 3.8. At the same time, the radiomics module was used to extract 93 radiomics features in these three VOIs, respectively. Next, we performed feature selection based on SHAP values. SHAP value could be used to measure the contribution of each feature in the model to the prediction results. By calculating SHAP, the ranking of the importance of features in the model could be obtained, and the top five important features are selected as the analysis index. Finally, according to the features selected for three VOI, three random forest models were established to evaluate the RAP level of patients. According to previous studies, RAP > 0.4 was considered to be an indicator of poor prognosis. In this study, 0.4 was used as the threshold value, and the dichotomous judgment was made with RAP. The whole research process is shown in Figure 1.

### Statistical analysis

Python 3.8 was used for the statistical analysis of the data. Numpy module and Pandas module were used for data operation and sorting, SHAP module was used for feature selection, RandomForestClassifier module of Sklearn was used for the model establishment, and the Matplotlib module is used for drawing. Continuous variables subject to normal distribution were expressed as the mean (M) ± SD, continuous variables not subject to normal distribution were expressed as the median and interquartile range (IQR), and categorical variables were expressed as the frequency and percentage. Accuracy, precision, recall, and F1 score were used to evaluate the performance of each model. The area under the receiver operator characteristic (ROC) curve was used in all four models to assess discrimination.

## Results

**Figure 2:**
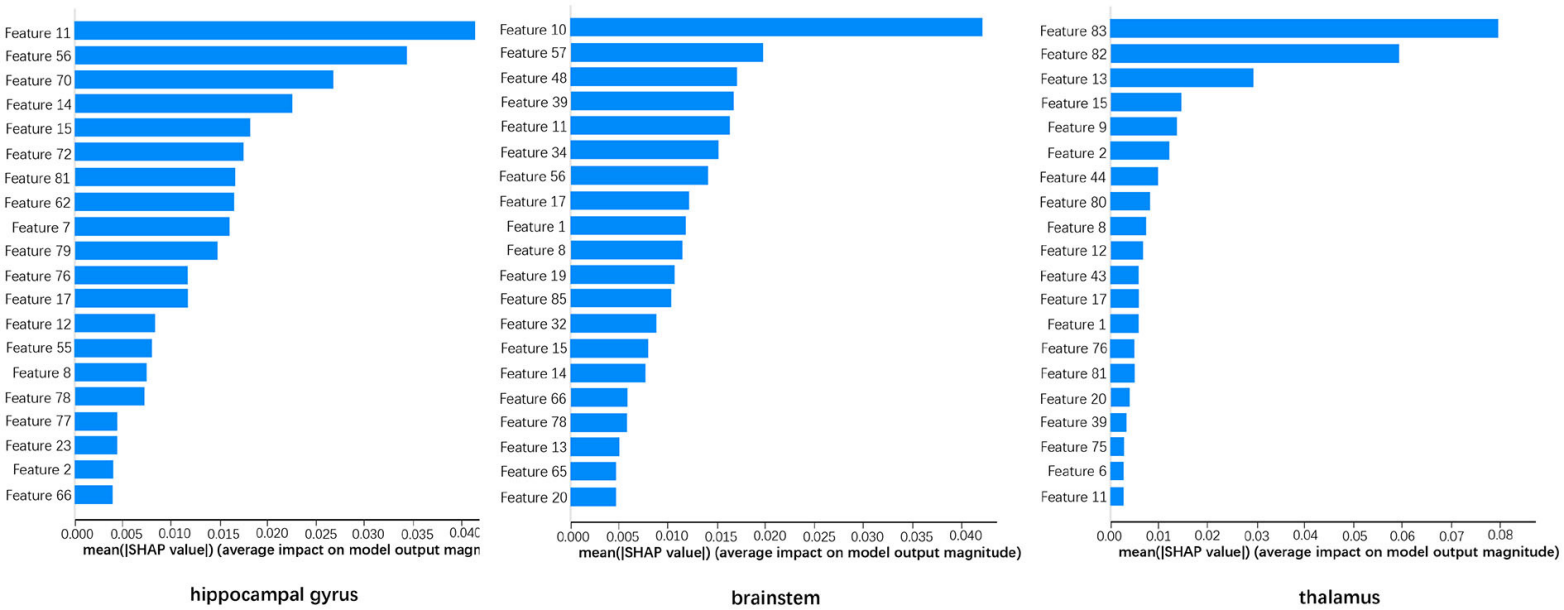
Feature importance based on SHAP value of hippocampal gyrus, brainstem, and thalamus.

From January 2019 to December 2020, a total of 36 patients with sTBI were included in this study, among which 25 were men (69.44%) and 11 were women (30.56%). Participants were between 18 and 65 years old, and the median age was 47 (IQR: 29–54) years old. The median Glasgow Coma Score (GCS) was 6 (IQR: 4–7) at the time of emergency admission; 27 patients (75%) had an abnormal light reflex in one or both pupils up-on arrival, among which postoperative pupil shrinkage was observed in 14 patients (51.85%). The order of feature importance based on the SHAP value is shown in Figure 2. Selected features of three VOI and collected RAP data was shown in Table 1.

**Table 1:** RAP and selected radiomics features.

	**Patients with sTBI (*n* = 36)**
RAP	0.24 (−0.02–0.51)
>0.4	9 (25%)
**Hippocampus (5 features)**	
Minimum	14 (12–16)
Root Mean Squared	35.5732 (34.0433–37.6606)
Skewness	−0.0832 (−0.1991–0.0401)
Small dependence low gray level emphasis	0.0096 (0.0079–0.0117)
Short run emphasis	0.4424 (0.3950–0.4832)
**Brainstem (5 features)**	
Median	31 (29–33)
Minimum	10 (8–12)
Maximum probability	0.7005 (0.5994–0.8129)
Gray level variance	0.1452 (0.0966–0.1861)
Gray level non-uniformity	125.6637 (118.9920–130.9465)
**Thalamus (5 features)**	
Mean	34.4850 (32.8150–35.5325)
Robust mean absolute deviation	3.7269 (3.3536–3.9609)
Skewness	−0.1189 (−0.2629–0.1033)
Size zone non-uniformity normalized	0.2234 (0.1878–0.2800)
Small area emphasis	0.3710 (0.2771–0.4710)

**Figure 3:**
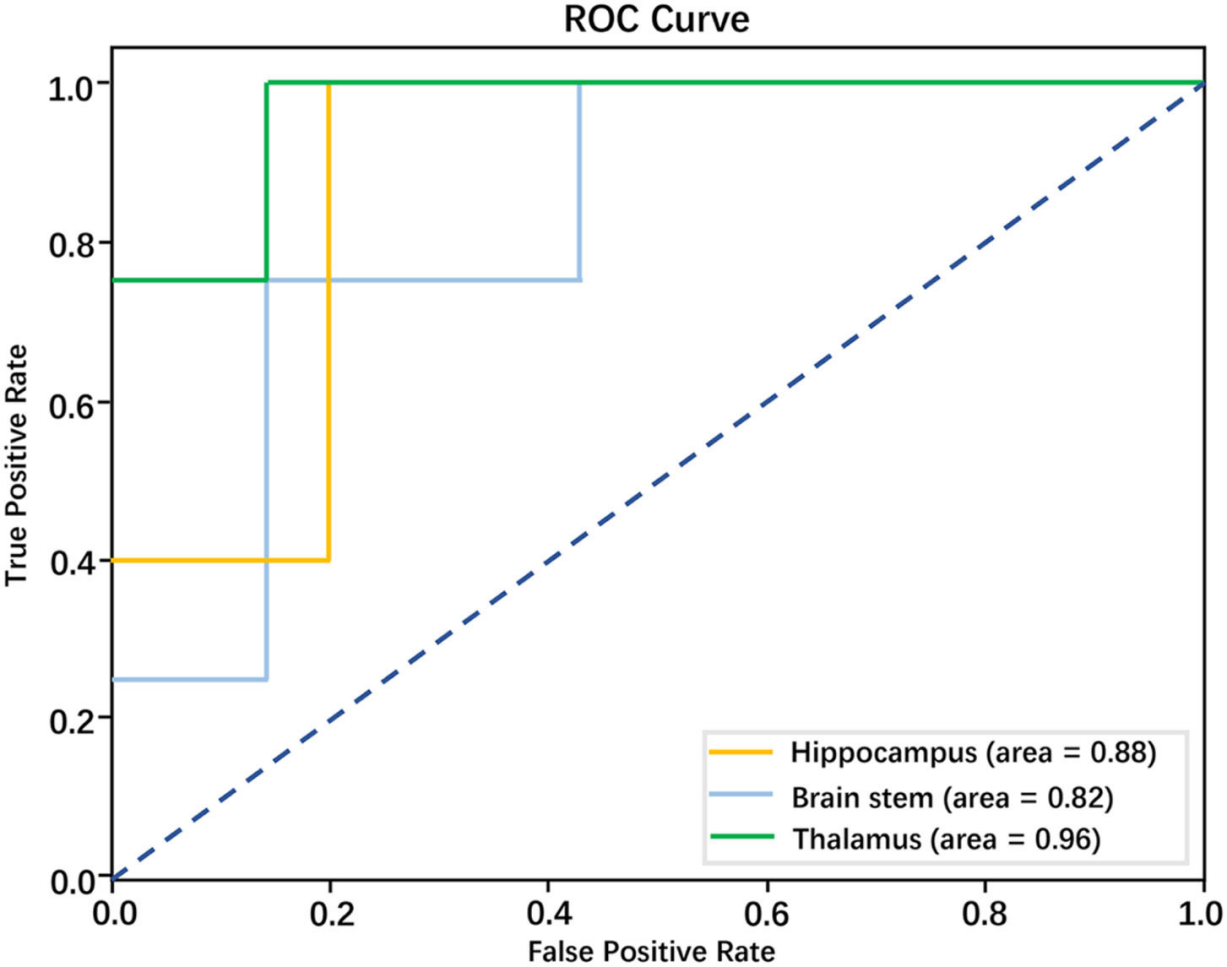
Performance of models based on radiomics features in discriminating RAP level; the AUCs for these models were 0.88, 0.82, and 0.96, respectively.

The optimal number of features for three predicting models of RAP was 5, respectively. The accuracy of predicting the model of the hippocampus was 77.78%, precision was 88.24%, the recall rate was 60%, the F1 score was 0.6, and AUC was 0.88. The accuracy of predicting the model of the brainstem was 63.64%, precision was 58.33%, the recall rate was 60%, the F1 score was.54, AUC was.82. The accuracy of predicting model of the thalamus was 81.82%, precision was 88.89%, the recall rate was 75%, the F1 score was 0.77, AUC was 0.96. These three models all had strong prediction ability and the model of the thalamus has the strongest prediction ability in discriminating RAP. The performance of three models in predicting RAP level is shown in Table 2 and Figure 3.

**Table 2:** Performance of models based on textural features and morphological features.

	**Hippocampus**	**Brainstem**	**Thalamus**
Accuracy	77.78%	63.64%	81.82%
Precision	88.24%	58.33%	88.89%
Recall	60.00%	55.36%	75.00%
F1 Score	0.60	0.54	0.77
AUC	0.88	0.82	0.96

## Discussion

This study retrospectively analyzed the CT images of patients with sTBI after operation treatment, conducted texture analysis, established three models to predict the RAP level according to the textural features obtained from selected morphological structure, and compared them with invasive ICP (I-ICP) monitoring. The results showed that three models all had strong prediction ability for the RAP level of patients with sTBI, the model of the thalamus has the strongest prediction ability in discriminating RAP. All of these results show CT radiomics has the potential to establish a method of non-invasive intracranial pressure (NI-ICP) monitoring.

Elevated ICP is the main pathophysiological change in patients with sTBI and the main cause of irreversible damage to brain function (7, 8). Early post-injury monitoring of sTBI patients with ICP and aggressive interventions based on changes are effective ways to reduce the mortality and disability rates (9, 10). Current ICP monitoring techniques are invasive and require the placement of ICP monitoring probes inside the patient's skull, which can lead to complications such as infection and bleeding (11, 12). Sometimes the time window for implantation of the ICP monitoring probe in the body does not meet the treatment needs, and monitoring has to be terminated to avoid the occurrence of infection, and the ICP monitoring needs for subsequent treatment cannot be met (13, 14). But, there is no established NI-ICP monitoring method that can serve as an alternative to the gold standards of I-ICP monitoring. To solve the above problems, Oliver et al. (15) evaluated a new method of NI-ICP monitoring performed using algorithms to determine ICP based on acoustic properties of the brain. Bernhard et al. (16) attempted non-invasive assessment of ICP by using cerebral blood flow velocity (CBFV) and arterial blood pressure (ABP) based on a mathematical model. Andersen et al. propose a method to assess ICP by using retinal arteriole and venule diameter ratio (A/V-ratio), A/V-ratio can be measured using fundus photography, they correlated changes in the intracranial pressure with the diameter of vessels of the retina (17). However, none of these programs have been widely promoted in clinical practice. Although ICP monitoring is more than half a century old and its application techniques have changed considerably in the process, it still has a lot of space for improvement in itself compared to the development of other scientific advances.

With the increased awareness of the concept of non-invasive, more and more protocols are being proposed, among which the CT image-based ICP evaluation is the most superior (18). CT is usually the front-line imaging approach in sTBI (18, 19). In the past, the analysis of CT images was limited to the interpretation of structural information, despite the widespread use of CT, their diagnostic accuracy for detection of elevated ICP and their correlation with ICP measurement are unknown. The analysis of CT texture features provides a perspective for establishing relevant assessment criteria and monitoring techniques (20). The morphological structural information alone is interpreted with a serious subjective bias (21). Texture features are quantifiable and can be analyzed to make abstract ICP monitoring data concrete, and provide a more objective basis for their interpretation. Here we analyzed texture features and then statistically integrated them to form a machine learning model to predict RAP in patients with sTBI. This is a complement to traditional machine learning models for predicting ICP and fills the gap in the direction of ICP-related parameter prediction by machine learning models.

The main damage after sTBI occurs is the destruction of important intracranial structures and functions (22). On top of these primary injuries, secondary injuries due to cerebral edema and hematoma further aggravate the damage to brain tissue and lead to deterioration of the condition. In our study, three important intracranial structures were selected for VOI extraction features to build models, in an attempt to fully explore the ability of CT imaging histology in machine learning to predict RAP. Among the three models, the thalamic model has the strongest predictive power, and we analyzed the reasons for this: (1) the thalamic structure is located in the deep part of the brain tissue, and its deformation and CT value changes are less subject to pressure changes, (2) the structural morphology of the thalamus is stable and is an ideal reservoir for texture feature extraction, and (3) the dense blood supply and blood flow in the thalamus are uniform. The above features are more universal in feature extraction and can fully reflect the internal changes of brain tissue. Building a machine learning model based on texture analysis for RAP capability is fully described in our study, while the feasibility of non-invasive ICP monitoring options can continue to be explored in future studies based on the above model. This provides potential options for the successful implementation of non-invasive ICP monitoring.

However, this study also had some limitations. Firstly, the number of cases is low. Secondly, studies focus on a single craniocerebral trauma type. Thirdly, retrospective patient past history should be considered in the study. The above issues will be addressed in subsequent studies, and further work will focus on expanding the capacity of the database and joint multicenter collaboration. The methods of CT impact histology quantification should be improved and more detailed and standardized criteria should be developed to fully take into account the impact of inter-case variation on the study, which should be assessed by a more sophisticated learning model.

## Conclusions

In summary, the machine learning model based on texture feature analysis was constructed to be able to predict the RAP of patients with sTBI. At the same time, the construction of such analysis and prediction models has application value and facilitation to achieving non-invasive ICP monitoring.

## Data availability statement

The original contributions presented in the study are included in the article/supplementary material, further inquiries can be directed to the corresponding author.

## Ethics statement

The studies involving human participants were reviewed and approved by Ethics Committee of Shanghai General Hospital. The patients/participants provided their written informed consent to participate in this study. The animal study was reviewed and approved by Ethics Committee of Shanghai General Hospital.

## Author contributions

JL, YS, and GG conceived the study. GG supervised and coordinated all aspects of the work and acquired the funding and administrated the project. JL and YS collected and analyzed the data and wrote the paper. All authors contributed to the article and approved the submitted version.

## Conflict of interest

The authors declare that the research was conducted in the absence of any commercial or financial relationships that could be construed as a potential conflict of interest.

## Publisher's note

All claims expressed in this article are solely those of the authors and do not necessarily represent those of their affiliated organizations, or those of the publisher, the editors and the reviewers. Any product that may be evaluated in this article, or claim that may be made by its manufacturer, is not guaranteed or endorsed by the publisher.
